# Deterministic, stochastic, and mean-field PDE models in neuroscience

**DOI:** 10.3389/fncom.2026.1762692

**Published:** 2026-04-20

**Authors:** Coşkun Çetin, Jose Roberto Castilho Piqueira, Burhaneddin İzgi, Ayse Peker-Dobie, Semra Ahmetolan, Murat Özkaya

**Affiliations:** 1 Department of Mathematics and Statistics, California State University, CSU, Sacramento, CA, United States; 2 Escola Politécnica, Universidade de São Paulo, São Paulo, Brazil; 3 Department of Mathematics Engineering, Faculty of Science and Letters, Istanbul Technical University, İstanbul, Türkiye; 4 Department of Business, Faculty of Political Sciences, Çanakkale Onsekiz Mart University, Çanakkale, Türkiye

**Keywords:** differential equation models, stochastic differential equations, computational neuroscience, stochastic neural dynamics, mean-field partial differential equations, Fokker-Planck equations, numerical methods

## Abstract

Large neuronal networks demonstrate complex dynamics across multiple scales, ranging from single-neuron excitability and spike-train variability to mesoscopic rhythms and whole-brain activity. Different types of differential equation models have been developed to comprehend these phenomena, connecting deterministic, stochastic, and mean-field descriptions. At the deterministic level, ordinary differential equation (ODE) models, including conductance-based neuron models, neural-mass systems, and whole-brain networks, summarize neural behavior through a reduced set of macroscopic variables. At the population level, mean-field partial differential equation (PDE) models such as Fokker-Planck, age-structured, kinetic, and neural field equations describe the evolution of probability or population densities over membrane-potentials, synaptic states, and other kinetic variables. These PDEs link single-neuron mechanisms to population-level activity and allow one to analyze bifurcations, oscillations and other collective patterns. Stochastic differential equation (SDE) models and their extensions that include jump-diffusion processes and stochastic PDEs (SPDEs) are widely used to describe random membrane fluctuations, irregular spike trains, synaptic plasticity and large-scale variability in neural activity. These stochastic models are also applied to neural data analysis, for example to quantify noise in electro-physiological recordings and to infer latent neural dynamics. Because variability and noise are central in neural systems, we devote more space to stochastic models but always relate them back to the surrounding ODE and PDE frameworks. This hierarchy of ODE, PDE, and SDE-SPDE models shows that the versatility of differential-equation-based approaches in neuroscience offers unified tools for multiscale modeling, neural signal processing, cognitive modeling, and the analysis of noisy neural systems. We also discuss some known numerical and computational approaches, especially for stochastic models and conclude by outlining open challenges, such as multiscale inference, control-oriented formulations and the integration of differential-equation models with modern machine-learning methods.

## 1 Introduction

Large neuronal networks show collective behavior that spans asynchronous irregular spiking, coherent oscillations and spatially structured activity patterns. Theoretical frameworks developed to explain these phenomena are mainly based on differential equations and can, on different scales, be grouped into three broad classes: deterministic ordinary differential equation (ODE) models, stochastic process-based descriptions, and mean-field partial differential equation (PDE) models arising in the large-network limit (Breakspear, 2017; Brunel, 2000; Carrillo and Roux, 2025; Gerstner et al., 2014). Our aim is to show how these three levels fit together into a multiscale mathematical picture that links single-neuron dynamics to whole-brain activity.

At the level of single-neurons and neural populations, ODE models form the classical backbone of computational neuroscience (Dayan and Abbott, 2001; Gerstner et al., 2014). Conductance-based models of Hodgkin-Huxley type describe the evolution of the membrane-potential together with gating variables that characterize the kinetics of the ion-channel, while reduced systems such as the FitzHugh-Nagumo and Morris-Lecar models capture key features of excitability and firing patterns in low-dimensional settings, making them suitable for phase-plane and bifurcation analysis (FitzHugh, 1961; Hodgkin and Huxley, 1990; Morris and Lecar, 1981; Nagumo et al., 1962). At the population scale, neural-mass models of Wilson-Cowan type, and their extensions to whole-brain settings, describe the interactions among excitatory and inhibitory populations, multistability, and large-scale oscillations in terms of a small number of macroscopic variables, such as firing rate or mean membrane-potential (Breakspear, 2017; Jansen and Rit, 1995; Wilson and Cowan, 1972). For broader reviews of mean-field and neural-mass approaches in computational neuroscience, see (Carrillo and Roux 2025); (Deco et al. 2008). For a complementary textbook treatment from an applied-mathematics perspective, including delays and stochasticity, see (Coombes and Wedgwood 2023). However, this deterministic skeleton is frequently extended to explicitly account for the stochastic nature of spike generation, synaptic noise, and network heterogeneity (Laing and Lord, 2009; Schwalger et al., 2017; Wallace et al., 2011).

This motivates a second layer of modeling in which ODE-based dynamics are generalized to stochastic processes. Noisy integrate-and-fire equations, stochastic conductance-based models, random spike-time models, Hawkes-type point-processes, and Markovian descriptions of synaptic and neuronal states allow one to incorporate both intrinsic fluctuations (such as conductance fluctuation of ion-channels) and extrinsic randomness (e.g., from the synaptic activity of other cells) as a natural generalization of deterministic models (Brunel, 2000; Carrillo and Roux, 2025; Daley and Vere-Jones, 2003; Gerstner and Kistler, 2002; Hawkes, 1971; Wallace et al., 2011). Stochastic differential equation (SDE) models as well as their extensions that include delay equations and jump-diffusion processes provide a flexible framework to model single-neuron and network dynamics in continuous time, bridging deterministic drift functions and random perturbations. They have been used to describe spike trains and membrane-potential fluctuations, to model synaptic plasticity and learning, and to capture large-scale neural variability in data such as electroencephalography (EEG) and related electrophysiological recordings (Ghorbanian et al., 2013; Tajmirriahi and Amini, 2021). More recently, the combination of SDE-based models with machine-learning tools and experimental data has contributed to our understanding of neural oscillations, decision making, and learning (ElGazzar and van Gerven, 2024; Holmes et al., 2005; René et al., 2020). At the same time, these approaches raise challenges in parameter estimation, model selection, and interpretation of fitted SDEs in biological terms (René et al., 2020). Such stochastic frameworks provide a microscopic description at the level of single-neurons or small networks and at the same time offer a natural starting point for the derivation of mean-field PDE models that describe the behavior of very large networks.

The third layer consists of mean-field PDE models, which do not track individual neurons but rather the evolution of population densities over appropriate state variables (Cáceres et al., 2011; Laing and Lord, 2009). Parabolic Fokker-Planck equations, such as network noisy leaky integrate-and-fire (NNLIF) models (sometimes called nonlinear noisy leaky integrate-and-fire models), describe how the distribution of membrane-potentials evolves under synaptic input and noise, and have been used to study phenomena such as blow-up, synchronization, and the stability of stationary states in recurrent networks (Cáceres et al., 2011; Cáceres and Perthame, 2014). Hyperbolic age- or time-elapsed-structured equations organize the population according to the time since the last spike and provide a natural framework to analyze the effects of refractoriness, adaptation, and delay (Kang et al., 2015; Pakdaman et al., 2013, 2014; Schwalger and Chizhov, 2019; Schwalger and Lindner, 2015). These age or time-elapsed structured equations are also widely known in the computational neuroscience literature as refractory-density equations (Aviel and Gerstner, 2006; Chizhov and Graham, 2007, 2008; Gerstner, 2000, 2001; Schwalger and Chizhov, 2019). Kinetic Fokker-Planck systems of voltage-conductance or FitzHugh-Nagumo type retain additional variables such as synaptic conductances or recovery variables, yielding mesoscopic models that preserve important biophysical detail (Mischler et al., 2016; Perthame and Salort, 2013). Stochastic partial differential equations (SPDEs), in particular neural field PDEs and spatially extended formulations, play a central role in the study of cortical waves, pattern formation, spatial aspects of brain dynamics as well as stochastic versions of time-elapsed PDEs (Carrillo et al., 2023, 2024; Schmutz et al., 2023; Schwalger et al., 2017). One can also check chapters 11–12 of (Laing and Lord 2009) for their numerical solutions.

The aim of this review is to present these three modeling layers not as separate topics but as parts of a coherent chain that runs from deterministic ODE models of single- neurons, through stochastic descriptions of spike generation and synaptic variability, to mean-field PDE models for large interacting populations. Because the stochastic layer provides the key bridge between microscopic neural variability and mean-field PDE descriptions, the stochastic material is developed in greater detail than the deterministic ODE and PDE sections. The remainder of the paper is organized as follows. Section 2 provides a concise overview of deterministic ODE models, summarizing the basic structure of single-neuron, neural-mass, and whole-brain dynamical systems. Section 3 introduces a brief theoretical background and some numerical solution methods for SDEs, and stochastic models, including SDE-based neuron and network descriptions, point-process and renewal models, and Markovian synaptic state models. Section 4 presents the main principles used to derive mean-field PDEs from microscopic stochastic network models. We also discuss Fokker-Planck, age-structured, kinetic, and neural field equations, combining modeling aspects, mathematical theory, and representative numerical results in this section. Finally, Section 5 highlights open problems and future research directions related to multiscale modeling, data assimilation, control, and the interplay between these mechanistic models and modern machine-learning approaches.

## 2 Deterministic ODE models in neuroscience: a brief overview

This section reviews the main classes of deterministic ODE models that have shaped theoretical and computational neuroscience. The goal is not to provide an exhaustive historical survey, but to illustrate the types of dynamics that arise at different scales and to highlight those structural features that will reappear in stochastic and PDE-based formulations.

### 2.1 Theoretical background

Deterministic neuronal models are naturally formulated as finite-dimensional dynamical systems. Given a state vector *x*(*t*) ∈ ℝ^*n*^ (for example, membrane-potential together with gating or recovery variables), a general ODE is given by


$$\begin{array}{l} ẋ\left(t\right)=F\left(x\left(t\right),t\right), \end{array}$$

where *F*:ℝ^*n*^×ℝ → ℝ^*n*^ is a vector field. In many neuroscience applications, *F* is autonomous *F*(*x, t*) = *F*(*x*), so the dynamics are fully determined by the current state. Under standard regularity assumptions on *F* (e.g., local Lipschitz continuity), the initial value problem *x*(0) = *x*_0_ defines a unique solution on some time interval and thus a flow Φ_*t*_(*x*_0_) on state space (Ermentrout and Terman, 2010; Izhikevich, 2007).

An equilibrium (or fixed point) is a state x^*^ such that *F*(x^*^) = 0. Linearizing the dynamics around x^*^ yields


$$\begin{array}{l} ẏ\left(t\right)=J\left(x^{*}\right)y\left(t\right),\;J\left(x^{*}\right)=DF\left(x^{*}\right), \end{array}$$

where *J*(x^*^) is the Jacobian matrix of *F* at x^*^. The eigenvalues of *J*(x^*^) determine the local behavior of the nonlinear system: If all eigenvalues have strictly negative real parts, x^*^ is (asymptotically) stable; if at least one eigenvalue has a positive real part, it is unstable. In low-dimension, phase-plane analysis provides a geometric picture of trajectories, allows one to classify excitable and oscillatory regimes, and facilitates the construction of bifurcation diagrams as the parameters vary (Ermentrout and Terman, 2010; Izhikevich, 2007). These tools will be used implicitly in our discussion of conductance-based and reduced single-neuron models.

Beyond fixed points, neuronal ODEs can exhibit limit cycles (periodic orbits), mixed-mode oscillations, bursting, and more complex attractors. Such behaviors often arise through local or global bifurcations (Hopf, saddle-node, homoclinic, etc.), in which qualitative and quantitative changes in long-time dynamics occur when parameters such as applied current or synaptic strength cross critical values. At population and whole-brain scales, these bifurcations underlie phenomena such as multistability between resting and active states, or transitions from asynchronous activity to rhythmic oscillations.

In many neural models, finite-transmission and processing-speed introduce explicit delays. Delay differential equations (DDEs) extend general ODEs by allowing the vector field to depend on past states,


$$\begin{array}{l} ẋ\left(t\right)=F(x\left(t\right),x\left(t-\tau_{1}\right),\cdots \;,x\left(t-\tau_{m}\right)), \end{array}$$

representing, for example, synaptic or axonal conduction delays. Such systems arise naturally in neural-mass and whole-brain models with long-range connectivity and can support rich dynamics, including delay-induced oscillations and complex transient behavior. In the present review, however, we do not pursue DDEs in detail and focus instead on ODE models without explicit delays, their stochastic extensions, and the corresponding mean-field PDE limits.

### 2.2 Conductance-based single-neuron models

At the level of a single-neuron, deterministic models describe the membrane-potential together with auxiliary variables representing ion-channel kinetics. The classical example is the Hodgkin-Huxley formalism, in which the membrane-potential *V*(*t*) satisfies a current-balance equation of the form


$$\begin{array}{l} C\dot{V}\left(t\right)=-\underset{k}{\sum }I_{k}(V\left(t\right),\text{x}\left(t\right))+I_{\text{syn}}\left(t\right)+I_{\text{ext}}\left(t\right), \end{array}$$ (1)

where *C* is the membrane capacitance, *I*_*k*_ denotes ionic currents (e.g., sodium, potassium, leak), *I*_syn_ is the total synaptic current and *I*_ext_ represents externally applied input (Dayan and Abbott, 2001; Gerstner et al., 2014; Hodgkin and Huxley, 1990). Each ionic current is typically written as


$$\begin{array}{l} I_{k}\left(V,\text{x}\right)={ḡ}_{k}m_{k}^{p_{k}}h_{k}^{q_{k}}\left(V-E_{k}\right), \end{array}$$

where ḡ_*k*_ is a maximal conductance, *E*_*k*_ is the reversal potential, and *m*_*k*_, *h*_*k*_ are gating variables that represent the fraction of open activation and inactivation gates for the channel type *k*. These gating variables satisfy first-order kinetics of the form


$$\begin{array}{l} {ṁ}_{k}=\alpha_{k}\left(V\right)(1-m_{k}(-\beta_{k}\left(V\right)m_{k}=\frac{m_{k,\infty }\left(V\right)-m_{k}}{\tau_{k}\left(V\right)}, \end{array}$$

with analogous expressions for *h*_*k*_. The vector **x**(*t*) collects all such gating variables. Together, Equation 1 and the gating equations define a nonlinear ODE system in which action potentials arise as emergent transients or oscillations.

Conductance-based models can be extended to include additional ion-channels, calcium and other intracellular variables, dendritic compartments, and detailed synaptic mechanisms. From a dynamical-systems viewpoint, they are relatively high-dimensional nonlinear ODE systems that can exhibit a variety of dynamical behaviors under parameter changes (Ermentrout and Terman, 2010; Izhikevich, 2007). These features will reappear, in aggregate form, at the population level.

Beyond reproducing action potentials, conductance-based models have been used to systematically classify different types of neuronal excitability and firing patterns. By varying maximal conductances, reversal potentials, or time constants, one can induce transitions between quiescence, tonic spiking, bursting and mixed-mode oscillations organized by local and global bifurcations in the underlying ODE system (Ermentrout and Terman, 2010; Izhikevich, 2007). This has led to robust classifications of neurons into, for example, type I and type II excitability according to the nature of the spike-onset bifurcation, with direct implications for how they synchronize in networks. Type I neurons can begin firing at arbitrarily low frequencies as the input current just crosses threshold (as in a saddle-node on invariant circle bifurcation), whereas type II neurons start firing at a finite, nonzero minimum frequency, typically through a Hopf bifurcation, and these differences have direct implications for how they synchronize in networks. Conductance-based models also serve as testbeds for parameter-estimation and model-reduction techniques, linking detailed ionic mechanisms to simplified descriptions that can be embedded into population and whole-brain models.

### 2.3 Reduced single-neuron models

To obtain simpler but dynamically faithful descriptions, reduced single-neuron models often approximate fast gating variables by their steady-state values and combine slower ones into a single recovery variable, leading to planar systems of the form


$$\begin{array}{l} \dot{V}=F\left(V\right)-w+I_{\text{ext}},\;ẇ=G\left(V,w\right), \end{array}$$

where *V* denotes the membrane-potential, *w* is a recovery variable, and *F, G* are nonlinear functions. The FitzHugh-Nagumo model (FitzHugh, 1961; Nagumo et al., 1962) and Morris-Lecar model (Morris and Lecar, 1981) are prototypical examples. Their low-dimensionality makes them ideal for phase-plane analysis: one can characterize excitability types, identify stable and unstable manifolds, and track qualitative changes in firing patterns via bifurcation diagrams (Ermentrout and Terman, 2010; Izhikevich, 2007). Classical dynamical systems analyses of neuronal excitability and bursting, especially phase-plane and fast-slow geometric methods developed by Rinzel and co-workers, provide important mathematical background for such reduced models (Rinzel and Huguet, 2013; Rinzel and Lee, 1987).

Another important family is provided by integrate-and-fire models. In their simplest form, the subthreshold dynamics of *V*(*t*) follow a linear ODE such as


$$\begin{array}{l} \dot{V}\left(t\right)=-\frac{1}{\tau_{m}}(V\left(t\right)-V_{\text{rest}})+\frac{1}{C}I_{\text{syn}}\left(t\right), \end{array}$$ (2)

where τ_*m*_ is the membrane time constant and *V*_rest_ is the resting potential. When *V*(*t*) reaches a fixed threshold *V*_th_, a spike is said to occur and the potential is instantaneously reset to a value *V*_reset_ (often followed by a refractory period). Mathematically, these are hybrid dynamical systems that combine continuous ODE flows with discrete events (Gerstner and Kistler, 2002). In later sections, noisy and networked versions of Equation 2 will serve as starting points for the derivation of Fokker-Planck and age-structured PDE models describing the evolution of membrane-potential or time-since-last-spike densities.

Reduced neuron models are frequently used as building blocks in network studies where analytic tractability is paramount. Planar FitzHugh-Nagumo or Morris-Lecar units coupled on lattices, random graphs, or small-world networks have been employed to investigate how local excitability interacts with connectivity to generate waves, synchrony, and cluster states. In the case of integrate-and-fire populations, their simplicity allows for large-scale simulations exploring how recurrent structure, heterogeneity, and synaptic delays shape emergent phenomena such as asynchronous irregular activity, oscillations, and related collective phenomena (Augustin et al., 2017; Brunel, 2000; Gerstner et al., 2014). Phase reductions of limit-cycle oscillator models provide yet another viewpoint, in which each neuron is reduced to a phase variable and coupling is encoded via phase response curves; this connects neuronal modeling to the broader theory of coupled oscillators and Kuramoto-type systems (Breakspear et al., 2010).

### 2.4 Neural-mass and rate-based ODE models

At a mesoscopic level, it is often convenient to aggregate the activity of many neurons into homogeneous populations and to describe their dynamics in terms of macroscopic variables such as average firing rate, average membrane-potential, or synaptic activity (Dayan and Abbott, 2001; Deco et al., 2008; Gerstner et al., 2014; Montbrió et al., 2015). This leads to neural-mass and rate-based models.

A paradigmatic example is the Wilson-Cowan system for interacting excitatory and inhibitory populations (Wilson and Cowan, 1972). Let *E*(*t*) and *I*(*t*) denote the activities (for example, firing rates) of the excitatory and inhibitory populations. Their evolution is given by


$$\begin{array}{l} \tau_{E}Ė\left(t\right)=-E\left(t\right)+\phi_{E}(w_{EE}E\left(t\right)-w_{EI}I\left(t\right)+I_{E}\left(t\right)),\\ \;\tau_{I}İ\left(t\right)=-I\left(t\right)+\phi_{I}(w_{IE}E\left(t\right)-w_{II}I\left(t\right)+I_{I}\left(t\right)) \end{array}$$

where τ_*E*_, τ_*I*_ are time constants, *w*_*ab*_ are coupling strengths between populations, *I*_*E*_, *I*_*I*_ are external inputs, and ϕ_*E*_, ϕ_*I*_ are typically sigmoidal gain functions. Variants of this model and related rate-based systems have been used extensively to study the emergence of oscillations, multistability, and pattern formation in cortical circuits (Ermentrout and Terman, 2010; Izhikevich, 2007; Wallace et al., 2011; Wilson and Cowan, 1972).

More elaborate neural-mass formulations, such as the Jansen-Rit model and its descendants, introduce additional state variables to represent postsynaptic potentials and synaptic currents (Jansen and Rit, 1995). Each population is then described by a small system of coupled ODEs with biophysically motivated parameters.

A notable recent development in mesoscopic population modeling is the emergence of exact mean-field, or next-generation, neural-mass descriptions for heterogeneous spiking networks (Coombes, 2023; Coombes and Wedgwood, 2023). In the quadratic integrate-and-fire/θ-neuron setting, these reductions connect microscopic spiking dynamics to closed macroscopic equations for quantities such as the firing rate, mean membrane potential, and, through Kuramoto/Ott-Antonsen-type descriptions, population synchrony (Devalle et al., 2017; Luke et al., 2013; Montbrió et al., 2015; Ott and Antonsen, 2008). This is conceptually important because firing rate is no longer treated as a purely phenomenological variable, but becomes dynamically linked to within-population synchrony. Extensions of this framework have also been used to describe fluctuation-driven population dynamics and synaptic shot noise in sparse balanced networks (Goldobin et al., 2025, 2021).

Neural-mass and rate-based models are often fitted directly to mesoscopic recordings, for example, EEG, MEG or local field potentials, to infer effective connectivity and to interpret pathological rhythms, thereby linking microscopic mechanisms to experimentally observed dynamics (Breakspear, 2017; Jansen and Rit, 1995). They have also been used to model cognitive operations such as working memory, attention, decision making and winner-take-all dynamics by exploiting multistability and nonlinear gain functions; more biophysically grounded recurrent circuit formulations have been especially influential in studies of working memory and related cognitive functions (Brunel and Wang, 2001; Compte et al., 2000). In this sense, neural-mass models occupy a central position between microscopic spiking descriptions and macroscopic imaging data: they incorporate biophysically interpretable parameters while remaining low-dimensional enough to permit systematic bifurcation analysis and parameter exploration (Augustin et al., 2017; Breakspear, 2017; Deco et al., 2008; Greven et al., 2026).

### 2.5 Whole-brain ODE networks

On the macroscopic, whole-brain scale, neural-mass or mean-field ODE models are often assigned to anatomically defined brain regions and coupled through structural connectivity matrices derived from diffusion-weighted MRI or tract-tracing data (Breakspear, 2017; Ritter et al., 2013). Denoting by *X*_*i*_(*t*) the state vector associated with the region *i* (e.g., local firing rates, mean membrane-potentials, and synaptic variables), one obtains a high-dimensional ODE network of the form


$$\begin{array}{l} {Ẋ}_{i}\left(t\right)=F(X_{i}\left(t\right))+\underset{j}{\sum }K_{ij}G(X_{j}\left(t-d_{ij}\right))+I_{i}\left(t\right), \end{array}$$ (3)

where *F* encapsulates the local neural-mass dynamics, *K*_*ij*_ is the structural connectivity from region *j* to region *i*, *d*_*ij*_ is an effective transmission delay and *I*_*i*_(*t*) denotes an external or noise-like input. Depending on the choice of local model and coupling, Equation 3 can reproduce key resting-state features, transitions between metastable patterns, and pathological regimes such as seizure-like activity (Breakspear, 2017; Deco et al., 2011; Naze et al., 2015).

From a mathematical perspective, Equation 3 defines a large system of coupled nonlinear ODEs, sometimes with delays, on a network. Questions of interest include the existence and stability of fixed points and limit cycles, the possibility of multistable regimes, the conditions for synchronization and partial synchrony, and the influence of network topology and delays on emergent dynamics (Breakspear, 2017).

Whole-brain models are typically constrained by structural connectivity matrices obtained from diffusion MRI or tract-tracing data, and their parameters are tuned to reproduce empirical measures such as functional connectivity, power spectra, or resting-state networks (Breakspear, 2017; Deco et al., 2011). Bifurcation analysis and numerical continuation have been used to identify parameter regimes that support realistic levels of metastability and dynamically switching network configurations, and to relate changes in coupling strength, conduction delays or regional excitability to shifts between healthy and pathological regimes, including seizure-like dynamics. These models thus provide a bridge between structural connectivity, dynamical systems theory, and clinical neuro-imaging, and they are natural candidates for control and data-assimilation approaches discussed later in the review.

### 2.6 Numerical exploration and bifurcation analysis

The deterministic ODE models reviewed so far are typically analyzed in combination with numerical simulation and bifurcation tools. For single-neuron models, direct time-stepping schemes such as forward or adaptive Runge-Kutta methods allow one to visualize trajectories, firing patterns, and responses to time-dependent inputs. In reduced planar systems of FitzHugh-Nagumo or Morris-Lecar type, phase-plane plots with nullclines, vector fields, and trajectories give a geometric picture of excitability and spike generation. They also provide a natural way to identify homoclinic orbits, limit cycles, and separatrices between different dynamical regimes (Ermentrout and Terman, 2010; Izhikevich, 2007).

In higher-dimensional conductance-based models, numerical continuation software (such as XPPAUT, AUTO, or MatCont) is routinely used to track equilibria and limit cycles as parameters vary, to locate Hopf, saddle-node, or homoclinic bifurcations, and to construct bifurcation diagrams in one- or two-parameter planes. These methods have been instrumental for classifying excitability and bursting mechanisms in Hodgkin-Huxley-type systems.

At the population and whole-brain scale, similar tools are applied to neural-mass and large-scale network models. The Wilson-Cowan and Jansen-Rit systems, and their whole-brain generalizations, are typically explored by combining direct numerical integration of coupled ODEs with continuation of steady states and periodic orbits. This allows one to chart regions of multistability, to identify bifurcation-induced transitions between asynchronous and rhythmic regimes, and to relate parameter changes (for example, in coupling strength or external input) to experimentally observed shifts between resting-state, oscillatory, and seizure-like activity (Breakspear, 2017; Jansen and Rit, 1995). In this sense, numerical bifurcation analysis forms a bridge between the abstract dynamical-systems framework introduced above and the phenomenology of realistic biophysical and whole-brain models.

## 3 Stochastic models of neural activity

Neural systems operate in an environment where information processing is affected by the electro-physiological properties of neurons and their bifurcation dynamics (Ermentrout and Terman, 2010; Izhikevich, 2007; Wallace et al., 2011) as well as the variability and noise of intrinsic cellular mechanisms and external input (Dumont et al., 2024; Fasoli, 2013; Laing and Lord, 2009; Pisarchik and Hramov, 2023; Saarinen et al., 2008). Although deterministic models provide valuable insight into the biophysical and network-level principles of neuronal dynamics, they often fail to capture the randomness observed in real neural data. Stochastic models offer a convenient framework for integrating these sources of randomness to model fluctuations in membrane-potentials, probabilistic spike generation, synaptic transmission variability, noisy limit cycles and large-scale network irregularities. As in many other applications of science and engineering, the simplest procedure to integrate randomness into deterministic models has been adding a Gaussian (or similar) white noise process to a deterministic dynamical system with a suitable intensity or standard deviation. For more complex models with autocorrelation functions, “colored noise” or other variations are incorporated into such models rather heuristically (Chizhov and Graham, 2007, 2008; Laing and Lord, 2009; Mao, 2011; Oksendal, 2013; Schwalger and Lindner, 2015). Both the microscopic origins and mesoscopic/macroscopic manifestations of neural variability can be described more rigorously via probabilistic models based on SDEs as well as their jump-diffusion, delay and SPDE versions (Cáceres et al., 2011; Carrillo et al., 2023, 2024; Dumont et al., 2024; Pietras et al., 2020; Schmutz et al., 2023). In this section, first a brief theoretical background and a review of commonly used numerical solution methods for SDEs are given. Then, several stochastic models in neuroscience are discussed in giving meaning to noisy experimental recordings, by highlighting the importance of randomness in the development of neural computation, population dynamics and parameter estimation.

### 3.1 Theoretical background

In this subsection, we introduce some definitions, notations, and known results for SDEs. We assume that the audience is familiar with probability distributions of random variables, conditional probability, covariance/variance, and probability spaces. Then, a stochastic process {*X*_*t*_:*t* ∈ *T*} is an indexed family of random variables in a given probability space, where each *X*_*t*_ represents the state of the system at time *t*.

**Markov property:** A stochastic process *X*_*t*_ is said to satisfy the Markov property (or it is a Markovian process) if for any time *t*_0_ ≥ 0 and any *n*-dimensional state space *S*, the conditional probability distribution of the process at a future time *t* ≥ *t*_0_ depends only on the state at time *t*_*n*_, and not on the history before that time. Formally, the Markov property is defined as: For any measurable subset *A* ⊂ *S* and *t*_0_ < *t*_1_ < ... < *t*_*n*_ < *t*,


$$\begin{array}{l} P\left(X_{t}\in A|X_{t_{0}}=x_{0},X_{t_{1}}=x_{1},...,X_{t_{n}}=x_{n}\right)=P\left(X_{t}\in A|X_{t_{n}}=x_{n}\right) \end{array}$$

where *X*_*t*_0__, *X*_*t*_1__, ..., *X*_*t*_*n*__ are the past states of the process up to time *t*_*n*_, and *X*_*t*_ is the state of the process at a future time *t* > *t*_*n*_.

**Wiener process**: A Wiener process (also called Brownian motion) is a Markovian stochastic process {*W*_*t*_ = *W*(*t*), *t* ≥ 0} that satisfies the following conditions:

- *W*(0) = 0 and *W*(*t*) − *W*(*s*) ~ *N*(0, *t* − *s*) for *t*>*s*, which means that the increments are Gaussian processes with mean 0 and variance *t* − *s*. In particular, *W*(*t*) ~ *N*(0, *t*) for all *t*>0
- For any sequence of time 0 ≤ *t*_1_ ≤ *t*_2_ ≤... ≤ *t*_*n*_, the increments *W*(*t*_2_) − *W*(*t*_1_), *W*(*t*_3_) − *W*(*t*_2_), ..., *W*(*t*_*n*_) − *W*(*t*_*n* − 1_) are independent random variables, which means that future increments are independent of the previous increments
- The process {*W*(*t*)} has continuous paths with probability one.

**Stochastic differential equations :** A stochastic differential equation (SDE) is a more general form of an ODE that involves a stochastic process to account for randomness in a dynamic variable *X*_*t*_. Here, our focus is on Ito-type SDE, which is of the Markovian form


$$\begin{array}{l} dX_{t}=f\left(t,X_{t}\right)dt+g\left(t,X_{t}\right)dW_{t}, \end{array}$$ (4)

where *X*_*t*_ is the random process at time *t*, *f*(*t, X*_*t*_) is the drift term representing the deterministic part of the system, *g*(*t, X*_*t*_) is the diffusion term denoting the stochastic part of the system, and *dW*_*t*_ is the "differential" term for a Wiener process *W*_*t*_. Equation 4 is usually written in an integral form which provides a more rigorous probabilistic representation. Moreover, its vector form can be used for higher dimensional state variables and several sources of noise (based on a multidimensional Brownian motion process). In that case the drift term is also a vector function and the diffusion term is a matrix function of a suitable size. However, such scalar or vector SDEs may not capture sudden or discontinuous changes/impulses directly. Then, the resulting discontinuous behavior of the random process can be modeled by adding a jump term to Equation 4 (Platen and Bruti-Liberati, 2010). These generalized processes are called jump-diffusion processes. Some examples and applications are given in subsection 3.3.

**Remark:** Note that a perturbed version of an ODE (e.g., the examples introduced in section 2) using a white noise process with a suitable deterministic intensity σ(*t*) can be described as an Ito-type SDE with the additive “noise” or diffusion term of the form σ(*t*)*dW*_*t*_. Then, the corresponding stochastic integral, $\int_{0}^{t}\sigma \left(s\right)\;dW_{s}$, is a Gaussian process with mean zero and variance $\int_{0}^{t}\sigma^{2}\left(s\right)ds$. The differential rules for Ito-type SDEs and their differentiable functions are governed by Ito stochastic calculus, more explicitly by a change-of-variable formula, called the Ito rule (Kloeden and Platen, 1992; Mao, 2011; Oksendal, 2013). Additionally, if jump-diffusion models are considered in the analyses, the jump-adapted approximation versions of these methods and their applications would take an important role (see İzgi, 2015, Chapter 4, Platen and Bruti-Liberati, 2010 and references therein).

### 3.2 Numerical solution methods for stochastic differential equations

Even though linear and some special nonlinear SDEs have explicit solutions, most nonlinear SDEs are solved using suitable numerical methods that include the Euler-Maruyama method and its several variants, as well as Milstein-type methods and other generalizations like Runge-Kutta versions. We present some common methods that are used in (or have the potential to improve) neuroscience models below.

**Euler-Maruyama method:** (Kloeden and Platen, 1992; Laing and Lord, 2009) The Euler-Maruyama (E-M) method approximates the solution to the SDE in discrete time steps. It is analogous to the Euler method for solving ODEs numerically, but this version also includes a stochastic component. If *X*(*t*) = *X*_*t*_ solves an SDE of the form


$$\begin{array}{l} dX_{t}=f\left(t,X_{t}\right)dt+g\left(t,X_{t}\right)dW_{t}, \end{array}$$

on an interval [0, *T*], we consider a partition of [0, *T*], 0 = *t*_0_<*t*_1_ < ... < *t*_*n*_ = *T*, and let *x*_*i*_ denote an approximation to *X*(*t*_*i*_) for each *i* = 0, 1, 2, ..., *n*. Then, starting with the initial condition *X*(0) = *x*_0_, which can be a constant or another random variable, the explicit (or forward) E-M method approximates this continuous-time process *X*(*t*) by its discrete-time E-M counterpart *x*_*i*_ via iterations of the form


$$\begin{array}{l} x_{i+1}=x_{i}+f\left(t_{i},x_{i}\right)\text{Δ}t+g\left(t_{i},x_{i}\right)\text{Δ}W_{i}, \end{array}$$

where Δ*W*_*i*_ is the random increment of the Wiener process with a Gaussian distribution. In particular, if a uniform partition is used with mesh size Δ*t*, then $\text{Δ}W_{i}\sim \sqrt{\text{Δ}t}N\left(0,1\right)$ can be used for simulating its paths via random numbers from the standard normal distribution. It is well known that the E-M procedure converges (strongly) with rate 0.5 as Δ*t* converges to zero if the drift coefficient satisfies a uniform Lipschitz condition and both the drift and diffusion terms have linear growth in the state variable *x* (Kloeden and Platen, 1992). Despite being simple and practical, this method is not suitable for more general equations, e.g., when a coefficient is only locally Lipschitz, or it has superlinear growth due to positive probability of sample path blow-ups and instability issues (İzgi and Çetin, 2018).

**Milstein Method:**(İzgi and Çetin, 2017; Kloeden and Platen, 1992) The Milstein method is another numerical scheme for solving SDEs, providing an improvement over the Euler-Maruyama method in terms of accuracy (improving the strong convergence rate to 1 under also smoothness assumptions on the diffusion coefficient). It is suitable for SDEs whose (differentiable) diffusion term is easy to differentiate with respect to the state variable. Given an SDE


$$\begin{array}{l} dX_{t}=f\left(t,X_{t}\right)dt+g\left(t,X_{t}\right)dW_{t}, \end{array}$$

the Milstein method improves upon the E-M method by adding an extra term involving the first partial derivative of the diffusion function *g*(*t, x*_*t*_). Then, a refined version of explicit E-M method that is called the explicit Milstein method has the following iterations at the discretized mesh points *t*_*i*_ with approximate solutions *x*_*i*_ to *X*(*t*_*i*_):


$$\begin{array}{l} x_{i+1}=x_{i}+f\left(t_{i},x_{i}\right)\text{Δ}t+g\left(t_{i},x_{i}\right)\text{Δ}W_{i}\\ \;+\frac{1}{2}g\left(t_{i},x_{i}\right)g^{\prime }\left(t_{i},x_{i}\right)\left({\left(\text{Δ}W_{i}\right)}^{2}-\text{Δ}t\right) \end{array}$$

where $g^{\prime }\left(t_{i},x_{i}\right)$ is the derivative of the diffusion term with respect to *x*_*t*_. Even though the explicit Milstein method is an improvement over the explicit E-M method, it suffers from the same divergence and instability phenomena when applied to only locally Lipschitz coefficients with superlinear growth, like stochastic FitzHugh-Nagumo models.

**Alternate Euler-type methods:** (İzgi and Çetin, 2018; Kloeden and Platen, 1992) In order to ensure convergence and stability of discretized numerical solutions, several alternate versions of E-M methods have been introduced. Some of them are modified to capture desirable properties of the actual solution (e.g., positivity, ergodicity etc.). The implicit E-M method for SDEs is a stochastic variant of the implicit Euler method that provides enhanced stability compared to the classical E-M and Milstein methods, especially for stiff problems with nonlinear drift terms over longer time periods [0, *T*]. It allows for larger time steps without numerical blow-up with the same strong convergence order of 0.5 (and weak convergence order of 1). For a given stochastic differential equation of the form in Equation 4, the (fully) implicit E-M method is given by the update rule below:


$$x_{i+1}=x_{i}+f\left(x_{i+1},t_{i+1}\right)\text{Δ}t+g\left(x_{i+1},t_{i+1}\right)\text{Δ}W_{i+1}.$$

A more practical version is drift-implicit E-M method, sometimes called partially implicit method, though the terms “partially implicit” and “semi-implicit” are used for several other types of numerical schemes including stochastic theta methods (for additively combining explicit and drift-implicit versions) and multiplicative combination of forward and backward values (*x*_*i*_ and *x*_*i*+1_ terms) in approximating the drift term. For an SDE of the form in Equation 4, iterations of the drift-implicit E-M method are given by


$$x_{i+1}=x_{i}+f\left(x_{i+1},t_{i+1}\right)\text{Δ}t+g\left(x_{i},t_{i}\right)\text{Δ}W_{i+1}$$

where the drift term *f*(*x*_*i*+1_, *t*_*i*+1_) is implicit, while the diffusion term *g*(*x*_*i*_, *t*_*i*_) is explicit.

The drift-implicit method provides superior stability and better computational efficiency for stiff SDEs compared with the explicit Euler and Milstein methods (requiring small Δ*t* steps and stronger assumptions on the drift term). Split-step methods also possess desirable convergence, stability, and long-term ergodicity properties in solving stiff SDEs. Some other methods that are known to converge under suitable monotonicity and polynomial growth conditions include truncated and tamed E-M methods. Each method has certain advantages depending on the type of nonlinearity, monotonicity, and growth conditions, step-size requirement, ease of implementation, computational efficiency, and empirical convergence behavior. For example, fully implicit, drift-implicit, and split-step methods have very strong stability properties, but they may require solving a nonlinear equation (e.g., via Newton method) at each iteration (İzgi and Çetin, 2018; Kloeden and Platen, 1992; Wang and Li, 2010). Truncated E-M algorithm restricts the growth of the coefficients to prevent the blow-up for solution processes or keep them positive if the solution is known to be positive. They provide improved stability and guarantee strong convergence of the method despite the fact that it involves a slight algorithmic bias in the numerical solution (Mao, 2015).

**Split-Step Euler method** (İzgi and Çetin, 2018; Wang and Li, 2010): Split-Step Euler (SSE) method approximates the solution of an SDE by separating its components, providing superior stability for stiff SDEs compared to explicit schemes. The SSE method first handles the stiff, deterministic drift part implicitly and then utilizes the stochastic diffusion part explicitly in a separate stage. This approach provides stability for the stiff components of the SDEs by allowing larger time steps for the solution process with the same convergence order of 0.5.

For SDE given in Equation 4, the drifting split-step Euler method is


$$\begin{array}{c} {\bar{x}}_{i}=x_{i}+f\left({\bar{x}}_{i},t_{i}\right)\text{Δ}t,\\ x_{i+1}={\bar{x}}_{i}+g\left({\bar{x}}_{i},t_{i}\right)\text{Δ}W_{i}. \end{array}$$

Moreover, if the first step is considered for the diffusion term instead of the drift, then the diffused version of the SSE method can be obtained, similarly. One drawback of SSE methods is that a nonlinear equation may need to be solved in the first stage at each iteration. One way to overcome this issue is to consider their semi-implicit versions for suitable drift functions (İzgi and Çetin, 2017, 2018). For example, consider a scalar logistic-type SDE of the form


$$\begin{array}{l} dX\left(t\right)=(AX\left(t\right)-\delta X^{r}\left(t\right)(dt+\sigma \left(X\left(t\right)\right)dW\left(t\right),\;0<t\leq T, \end{array}$$

with the initial condition *X*(0) = *x*_0_, where *r* = 2 or an odd positive integer *r* ≥ 3, δ ≥ 0, *A* ∈ ℝ, and σ(·) satisfies a linear growth condition.

Then, the semi-implicit split-step (SISS) method for this scalar SDE focuses on approximating the term *a*(*y*) = *Ay* − δ*y*^*r*^ with $\rho_{1}\left(x,y\right)=Ay-\delta yx^{r-1}$, or with $\rho_{2}\left(x,y\right)=Ax-\delta yx^{r-1}$. Starting with the former choice, if we solve *y* = *x*+ρ_1_(*x, y*)Δ for *y*, then we obtain the expressions


$$\begin{array}{l} f^{\text{Δ}}\left(x\right)=\frac{x}{1+\text{Δ}\left(\delta x^{r-1}-A\right)},\;a^{\text{Δ}}\left(x\right)=\frac{a\left(x\right)}{1-\text{Δ}\left(A-\delta x^{r-1}\right)}, \end{array}$$

where *f*^Δ^(*x*) = *x*+Δ*a*^Δ^(*x*). These functions are well-defined if δ*x*^*r* − 1^ − *A* ≥ 0 or if Δ is sufficiently small. It is easy to verify that *a*^Δ^(*x*) converges uniformly to *a*(*x*) in compact sets as Δ → 0 (see İzgi and Çetin, 2018, 2021).

A similar result is obtained if the latter approximation ρ_2_(*x, y*) is used. Then, several alternative versions of the SISS methods can be constructed (İzgi and Çetin, 2018). A simple version uses the following (now explicit) iterations: $x_{i+1}=f^{\text{Δ}}\left(x_{i}\right)+\sigma \left(x_{i}\right)\text{Δ}W_{i+1}$ where *f*^Δ^(*x*) and *a*^Δ^(*x*) are as above. So, this SISS version corresponds to the E-M discretizations applied to an SDE that has a new drift term *a*^Δ^(*x*), instead of *a*(*x*). On the other hand, one may consider the Milstein-type SISS methods in İzgi and Çetin (2019), whose order of strong convergence is 1, as an extension of the Euler-type SISS methods.

### 3.3 Existing work of SDEs on mathematical neuroscience

In this section, we provide an integrated overview of stochastic models in neuroscience during the last few decades, highlighting general trends, contributions, strengths, and limitations of existing research in mathematical neuroscience. Incorporation of randomness to neural models in literature has involved Brownian motion (from white noise), Ornstein-Uhlenbeck process or Langevin equation (from colored noise), Poisson process (from jumps and arrival times of inputs), Boltzmann machine (for binary neuron models), and Markov chain models (with suitable transition probabilities and discretized states), among others (Burkitt, 2001; Dayan and Abbott, 2001; Fasoli, 2013; Greven et al., 2026; Pietras et al., 2020; Schwalger et al., 2017; Wallace et al., 2011; Wegman and Habib, 1992; Yasue et al., 1988). However, our focus in this review is mainly on models which are based on Ito-type SDEs, Poisson processes and jump-diffusion models.

In order to model the kinetics of the relative membrane-potential *X*(*t*) of *N* neurons in a nerve system with mutual electric interactions via neural connections, an *N*-dimensional Wiener process *W*(*t*) and a vector SDE of the form


$$\begin{array}{l} dX\left(t\right)=A\left(t\right)dt+\sqrt{v}dW\left(t\right), \end{array}$$

are introduced in (Yasue et al. 1988). In this model, each component *x*_*i*_(*t*) has a drift term, *A*_*i*_(*t*), which represents the mean forward current of the neuron *i*, as the total electric current flowing into its membrane. They use the least action principle and variational methods to derive a fundamental equation, which is a complex neural wave equation. The proposed model captures fundamental perceptual processes and memory mechanisms.

The sources, forms, and functionality of noise/uncertainty terms in neurodynamics have been extensively discussed in the literature. The noisy input from presynaptic neurons may regulate firing, explain subthreshold vs. suprathreshold activities, break up phase locking (or produce a stochastic phase locking called skipping), and improve encoding of incoming signals via stochastic resonance. Noise is present at all stages of sensory processing and significantly affects variability in both neural and behavioral responses. It may also contribute to balancing energy cost and reaction times in neural design. Thus, noise is not just a nuisance term, but a helpful part of how the brain processes and encodes information and makes decisions (Fasoli, 2013; Laing and Lord, 2009; Pisarchik and Hramov, 2023). The (constructive and more general) regulation effect of noise to enhance firing is known as coherence resonance (Deco and Schürmann, 1998; Laing and Lord, 2009; Pisarchik and Hramov, 2023), while anticoherence resonance refers to the diminishing regularity effects of noise intensity (Pisarchik and Hramov, 2023).

In order to study how noise can enhance information transfer in central neurons through stochastic resonance, a stochastic integrate-and-fire model for the subthreshold membrane-potential and its jump-diffusion version is adopted in (Deco and Schürmann 1998). This approach combines an Ornstein-Uhlenbeck SDE with a Poisson-distributed input spike train (from somatic synapses) as a jump-diffusion model:


$$dV(t)=\left(-\frac{V(t)}{\tau }+\mu \right)dt+\sigma dW(t)+\omega dS(t),$$

where τ denotes the decay of the membrane-potential in the absence of the input signal, ω represents soma-synaptic-strength, and *S*(*t*) is a homogeneous Poisson process as a train of Dirac delta functions. More explicitly, *S*(*t*) satisfies $\frac{dS\left(t\right)}{dt}=\underset{i}{\sum }\delta \left(t-t_{i}\right)$ over all spike times *t*_*i*_ which are governed by a Poisson process with fixed intensity λ (Deco and Schürmann, 1998). Jump-diffusion models also appear in the applications of synaptic plasticity. For example, (Robert and Vignoud 2021) used an SDE for the time evolution of the post-synaptic membrane-potential, and the neuron spikes are modeled by an inhomogeneous Poisson process.

When the average overall synaptic input is large, the corresponding Poisson process can be approximated by a suitable Gaussian process. Then, the synaptic current dynamics with “white noise” would follow an Ornstein-Uhlenbeck process. Consequently, this uncertainty appears on the voltage dynamics in the form of “colored noise”. An application to the stochastic FitzHugh-Nagumo model with additive and periodic stochastic forcing can be written in the following SDE form (with a slight change of notation):


$$\begin{array}{l} dV(t)=\left(bV(V-0.5)(1-V)-cw(t)+d\sin(ft)+I(t)\right)dt\\ \;+\sigma dZ(t), \end{array}$$

where *w*(*t*) is a slow recovery variable (which depends on *V* in a linear ODE), *I*(*t*) is the bias current, *f* is the forcing frequency and *Z*(*t*) is an Ornstein-Uhlenbeck process. For a summary of these ideas and references, one can check (Laing and Lord, 2009, Chapter 4). Note that such a system of nonlinear equations can be efficiently solved via the SISS methods described in subsection 3.2 of this manuscript.

By focusing on two independent Poisson processes (for excitatory and inhibitory postsynaptic potentials, respectively), the behavior of balanced neurons with reversal potential is analyzed in (Burkitt 2001) using a stochastic version of the leaky integrate-and-fire (LIF) model. This stochastic line also connects to classical first-passage-time and diffusion-based analyses of leaky integrate-and-fire models (Ditlevsen and Lansky, 2007; Lansky and Ditlevsen, 2008). Related analytical work has further developed fluctuation-dissipation and cross-correlation-response relations for spiking systems under diffusive and shot-noise drive (Lindner, 2022; Stubenrauch and Lindner, 2024). For balanced neurons that receive periodic input, the spiking rate is modeled with an inhomogeneous Poisson process, and the relationships between the timing of these periodic synaptic inputs and the output spikes are studied. Using an Ito-type SDE model for LIF neurons, rapid random fluctuations (RRF) in neurons are studied in (Hong et al. 2016) to explain how brain signals propagate. The model captures RRF in neuronal membrane potentials and offers analytical support for the presence of a force that drives signal movement in the brain.

By adding Brownian noise to the Hodgkin-Huxley model, an Ito-type SDE is used to model voltage-gated ion-channel behavior in (Saarinen et al. 2008) with a focus on cerebellar granule cells (which are small excitatory neurons). They form an SDE model with the activation and inactivation of six different ionic conductances:


$$\begin{array}{l} dX\left(t\right)=\left(\alpha_{x}\left(V_{m}\right)\left(1-X\left(t\right)\right)-\beta_{x}\left(V_{m}\right)X\left(t\right)\right)dt+\sigma dW, \end{array}$$

where *X*(*t*) represents the gating variable for the ion-channel type in question, α_*x*_ and β_*x*_ are the rate functions of activation or inactivation processes, respectively, and *W* is the Brownian motion. They emphasize the role of the SDE approach for more accurately and efficiently modeling the random behavior of neurons and ion-channels, compared to Markov chain and deterministic models. They reproduce irregular firing observations of cerebellar granule cells more realistically and efficiently than previous stochastic methods by also using the expectation-maximization algorithm. This method has the potential to investigate intricate neural network dynamics in the cerebellum and other neuron types as well (Saarinen et al., 2008).

Another application of stochastic models to improve deterministic methods is modeling cerebrospinal fluid (CSF) flow and intracranial pressure fluctuations observed in clinical data. For example, a stochastic version of the Marmarou model is developed in (Raman 2011) by introducing the following Ito-type SDE for the dynamic flow of CSF:


$$dp=\left\{EpI(t)-\frac{Ep(p-p_{b})}{R}\right\}dt+\sigma EpdW,$$

where *p* is the CSF pressure, *p*_*b*_ is the basline pressure, *E* is the cerebral elasticity and *I* is the rate of external volume addition. This SDE has an explicit analytical solution (as a stochastic Verhulst equation), but its more complex versions and similar quadratic models [like the quadratic integrate-and-fire models in (Laing and Lord 2009)] can be solved using the stable numerical methods discussed in Subsection 3.2. One of the key findings of this study is that when noise reaches a certain level, the probabilities change quickly based on how easily CSF flows and how strong the noise is (Raman, 2011).

By utilizing perturbative field-theoretic and path integral methods, the moments of commonly used SDEs such as FitzHugh-Nagumo and Ornstein-Uhlenbeck models are studied in (Chow and Buice 2015). While these methods are complex, they are powerful tools in mathematical neuroscience and can also be applied to study larger systems such as networks of interacting neurons or systems with fixed disorder.

Recent literature and dissertations in stochastic neural dynamics also include models with multiple variables usually coupled together to explain how one or more brain regions interact, how to predict spiking behavior of neurons, or how the membrane potential dynamics are related to intracellular movement. For example, the following (reduced) collection of coupled nonlinear SDEs is proposed in (Deco et al. 2014) for the global brain dynamics for the excitatory (E) and inhibitory (I) variables:


$$\begin{array}{l} \;I_{i}^{\left(E\right)}=W_{E}I_{0}+w_{+}J_{NMDA}S_{i}^{\left(E\right)}+GJ_{NMDA}\sum_{j}C_{ij}S_{j}^{\left(E\right)}-J_{i}S_{i}^{\left(I\right)}\\ \;+I_{external}\\ \;I_{i}^{\left(I\right)}=W_{I}I_{0}+J_{NMDA}S_{i}^{\left(E\right)}-S_{i}^{\left(I\right)}+\lambda GJ_{NMDA}\sum_{j}C_{ij}S_{j}^{\left(E\right)}\\ \;r_{i}^{\left(I\right)}=H^{\left(I\right)}(I_{i}^{\left(I\right)})=\frac{\alpha_{I}I_{i}^{\left(I\right)}-b_{I}}{1-exp(-d_{I}(a_{I}I_{i}^{\left(I\right)}-b_{I}))}\\ \frac{dS_{i}^{\left(E\right)}(t)}{dt}=-\frac{S_{i}^{\left(E\right)}}{\tau_{E}}+(1-S_{i}^{\left(E)\right)}\gamma r_{i}^{\left(E\right)}+\sigma v_{i}(t))\\ \frac{dS_{i}^{\left(I\right)}(t)}{dt}=-\frac{S_{i}^{\left(I\right)}}{\tau_{I}}+r_{i}^{\left(I\right)}+\sigma v_{i}(t), \end{array}$$

where $r_{i}^{\left(E,I\right)}$ denotes the firing rate of the excitatory (E) or inhibitory (I) population in brain area *i*, $S_{i}^{\left(E,I\right)}$ is the average excitatory or inhibitory synaptic gating variable at area *i*, and $I_{i}^{\left(E,I\right)}$ represents the corresponding input current to the excitatory or inhibitory variable, respectively. Their approach simplifies a high-dimensional system of neurons interacting randomly by treating groups of elements as independent entities in a dynamic-mean-field model (Deco et al., 2014).

By expanding the Hodgkin-Huxley neuron model to a system of four SDEs, (Tierney 2024) investigates the spiking behavior of a neuron to provide a more realistic understanding of the dynamics of membrane potential (voltage, *V*) as well as the gating variables (*m, n, h*) at a given time in the ion channel:


$$\begin{array}{l} \;dV=\left[\left(I-\left(I_{N\alpha }+I_{K}+I_{L}\right)\right)/C\right]dt+\sigma_{V}dW^{1}\left(t\right)\\ dm=\left[\alpha_{m}\left(V\right)\left(1-m\right)-\beta_{m}\left(V\right)m\right]dt+\sigma_{m}dW^{2}\left(t\right)\\ \;dh=\left[\alpha_{h}\left(V\right)\left(1-h\right)-\beta_{h}\left(V\right)h\right]dt+\sigma_{h}dW^{3}\left(t\right)\\ \;dn=\left[\alpha_{n}\left(V\right)\left(1-n\right)-\beta_{n}\left(V\right)n\right]dt+\sigma_{n}dW^{4}\left(t\right) \end{array}$$

where *m* and *n* represent the proportions of sodium or potassium gates open, respectively, and *h* refers to proportion for sodium ion channel closed. Moreover, σ_*V*_, σ_*m*_, σ_*h*_, and σ_*n*_ denote the diffusion coefficients on each of the four states of the model. The model is solved via the E-M method, and an ensemble Kalman filter method is implemented for data assimilation to describe how the stochasticity influences a neuron's behavior (Tierney, 2024).

A non-linear stochastic model of nine variables is introduced in Ðorđević et al. (2023) for dopamine dynamics in rat striatum. The model captures synthesis, storage, release, uptake, and metabolism, whose noise terms are driven by a nine-dimensional Brownian motion process. The article also proves the existence, uniqueness, and positivity of solutions and presents boundaries for the moments of the SDEs. They use a positivity-preserving and convergent implicit numerical method to simulate coordinate processes and provide visualizations.

A multidimensional stochastic model is considered in (Ditlevsen and Samson 2016) to estimate biophysical parameters of neuronal membrane-potential dynamics from intracellular recordings. The model uses an *m*-dimensional Wiener process *W*_*t*_, a *p*-dimensional parameter vector θ, and a *d*-dimensional process of the form *X*_*t*_ = (*V*_*t*_, *Y*_*t*_) in the Ito-type SDE


$$\begin{array}{l} dX_{t}=b\left(X_{t};\theta \right)dt+\text{Σ}\left(X_{t};\theta \right)dW_{t}, \end{array}$$ (5)

where *V*_*t*_ denotes the membrane-potential, and *Y*_*t*_ represents the *d* − 1 dimensional vector of unobserved variables, including gating variables and the proportion of open ion channels (for a specific ion) or specific synaptic input. In Equation 5, the expressions *b* and Σ are the corresponding vector drift and matrix diffusion functions, respectively. The main challenge is parameter estimation, as only the membrane-potential is directly observed, making it harder to infer hidden states from these partially observed complex models.

Many research papers study brain damage/injuries and diseases, including seizures and Alzheimer's disease, via stochastic models and their numerical solutions, occasionally with the aid of machine learning methods. For example, a stochastic brain network model is presented in Jirsa et al. (2017) for brain interventions by incorporating individual structural data, epileptogenic zones, and MRI lesions. Using high-performance computing and simulations of SDEs via the E-M method, they conduct systematic parameter exploration and model validation for the development of tailored therapeutic strategies. A high-order linear SDE dynamic system in a *d*-dimensional space is used in (Chen et al. 2017) to model and optimize the movement trajectory (selected by the central nervous system) of the human arm. By extending the integrate-and-fire model (for stochastic neuron activity) and employing a stochastic control method, analytical solutions for the optimal trajectory, velocity, and variance are explicitly derived in this linear model, and a three-dimensional example is provided.

By utilizing advanced connectometry and a stochastic model, Sinha et al. (2019) studies significant white-matter changes in patients with idiopathic generalized epilepsy (IGE), including damage to the cingulum, fornix, and superior longitudinal fasciculus, as well as stronger thalamo-cortical tract integrity. Incorporating these findings into a computational model, the study suggests that enhanced cortico-reticular and impaired cortico-cortical connections drive seizure-like dynamics, highlighting impaired cortical-to-thalamic connectivity as a potential therapeutic target. Seizure transitions were simulated using an SDE model with a noise term, numerically solved via the E-M method with a very small step size.

A new EEG signal modeling approach using SDE with self-similar fractional Lévy stable processes (the innovation model) is introduced in (Tajmirriahi and Amini 2021) to extract discriminative features for seizure detection. By using a scale-invariant fractional derivative and fitting a probability distribution to the derived signals, the features are then used to train a support vector machine (SVM) classifier. This approach achieves very high classification accuracy (of more than 99 percent) in distinguishing between healthy and epileptic EEG segments. The simple and accurate method is also computationally efficient as it does not require signal decomposition. Another study that employs an innovation process and machine learning methods (SVM, K-nearest neighbor, and random forest) for high classification accuracy is the SDE model of (Raisi-Nafchi et al. 2025b), which is applied to post-contrast T1-weighted MRI images for grading astrocytomas (a type of brain tumors) before surgery. The model utilizes a fractional Laplacian filter to capture specific properties of the MRI data and then feeds the results into the classified grades. The proposed model succeeds in obtaining high accuracy (98.49%) using a two-dimensional SVM, and shows great promise for improving tumor grading (Raisi-Nafchi et al., 2025b). These authors also applied SDEs along with a statistical modeling framework to analyze MRI images and predict the isocitrate dehydrogenase mutation status in grade IV gliomas (Raisi-Nafchi et al., 2025a).

A novel stochastic model is introduced in (MacIver and Shaheen 2024) by adding Brownian diffusion to a deterministic reaction-diffusion model used in biological invasion applications. By integrating ideas from graph theory and Bayesian statistics, this model is used to simulate the propagation of misfolded proteins in Alzheimer's disease, a neuro-degenerative disorder characterized by cognitive decline. The corresponding SDE formulation is given by


$$dc_{i}=\left(-\sum_{j=1}^{N}L_{ij}c_{j}+\alpha c_{i}[1-c_{i}]\right)dt+\sigma^{2}c_{i}dW(t),$$

where *c*_*i*_ denotes the misfolded protein concentration in node *i*, and α represents the conversion rate. This stochastic version contributes to more realistic simulations of the disease and captures the variability in misfolded protein concentration across different brain regions over time.

By adding Brownian noise to a biologically plausible deterministic (Izhikevich) model and utilizing machine-learning techniques, a neural SDE is proposed by (Sato et al. 2025) for modeling heterogeneous spike patterns, designing external current and controlling spike-timing. External currents are iteratively trained to minimize both firing mismatches and timing errors using stochastic gradient descent and back-propagation techniques. Simulations indicate successful spike control including regular spiking, bursting, and fast spiking patterns.

A stochastic model based on Langevin equations and Fokker-Planck equation is introduced in (Staii 2025) to describe random axonal motion, connect the drift to a biophysical actin-myosin-clutch model, and investigate the stability for estimating the fluctuation spectra and oscillatory regimes.

From a diffusive approximation of the mean-field limit of a system of SDEs that model the collective behavior of an ensemble of neurons, (Carrillo et al. 2013) examined the global existence of classical solutions to the initial boundary-value problem of a nonlinear parabolic equation. The study derives a nonlocal Fokker-Planck equation with nonlinear dependence on the coefficients that includes the probability flux across the boundary. Then the Fokker-Planck equation with nonlinear boundary conditions is transformed into a Stefan-like free boundary problem with a Dirac-delta source term.

Some other multidimensional stochastic (integrate-and-fire) neuron models discuss spike-triggered adaptation in non-equilibrium settings (Schwalger and Lindner, 2015), subthreshold resonance under weak and high noise settings (Brunel et al., 2003), colored noise for temporally correlated spike inputs (Schwalger et al., 2015), noisy limit cycles and quasi-cycles (Wallace et al., 2011), and dimension reduction techniques that include spectral decomposition of the corresponding Fokker-Planck operator (Augustin et al., 2017).

In conclusion, these studies highlight significant recent advances for stochastic models in the neurosciences. The integration of stochasticity in neuroscience research provides a more realistic approach, enabling models to better explain the behavior of neurons and brain activity.

Modeling the interaction of a large population of neurons becomes challenging as the complexity of the neural system grows. Although SDEs provide better insight into the dynamics of a single-neuron or a neural network, it is computationally difficult to use a very large system of SDEs in modeling the entire brain. To overcome this issue, the mean-field PDEs are utilized. The mean-field PDEs model large-scale neural interactions and provide a mesoscopic/macroscopic description of them. These equations connect the gaps between the models for individual neurons and those for collective groups of neurons observed in neural systems. Their details are discussed in the next section.

## 4 Mean-field PDE models in neuroscience

Mean-field PDE models provide mesoscopic/macroscopic descriptions of large neuronal networks by following probability or population densities over suitable state variables instead of tracking individual neurons. From a modeling viewpoint, they arise as limits of large interacting stochastic systems and can often be interpreted as nonlinear Fokker-Planck or transport equations of McKean-Vlasov type (Fasoli, 2013); see also (Carrillo and Roux 2025) for a recent comprehensive survey of such models. Related mesoscopic reductions linking spiking-network dynamics to population-level descriptions include the dynamic mean-field approach of Mattia and Del Giudice, spike-to-rate reductions such as those of Ostojic and Brunel, and fluctuation-driven population reductions developed more recently by (Goldobin et al. 2021); (Mattia and Del Giudice 2002, 2004); (Ostojic and Brunel 2011).

In this section, we first recall the mean-field construction in a unified framework, and then discuss four PDE families that have emerged in neuroscience: parabolic Fokker-Planck equations for membrane-potential or rate variables (Section 4.2), hyperbolic age- or time-elapsed structured equations (Section 4.3), kinetic Fokker-Planck systems for voltage-conductance and FitzHugh-Nagumo-type dynamics (Section 4.4), and stochastic neural field PDEs for spatially extended cortical activity (Section 4.5).

### 4.1 Theoretical background: from microscopic networks to mean-field PDEs

Consider a network of *N* neurons. For each neuron *i* ∈ {1, …, *N*} we denote by *Z*_*i*_(*t*) its state at time *t*, taking values in a space *E* (e.g., *E* = ℝ for a membrane-potential, *E* = ℝ^2^ for a voltage-recovery pair, or *E* = ℝ_+_×ℝ for an age-potential pair). A broad class of interacting stochastic models can be written as


$$\begin{array}{l} dZ_{i}\left(t\right)=b(Z_{i}\left(t\right),\mu_{t}^{N})dt+\sigma (Z_{i}\left(t\right),\mu_{t}^{N})dW_{i}\left(t\right)+dJ_{i}\left(t\right),\\ \;i=1,\ldots ,N, \end{array}$$

where $\mu_{t}^{N}=\frac{1}{N}\overset{N}{\underset{j=1}{\sum }}\delta_{Z_{j}\left(t\right)}$ is the empirical measure of the network, *b* and σ are drift and diffusion coefficients, *W*_*i*_'s are independent Brownian motions (or other noise sources), and *J*_*i*_ represents jump terms such as spikes and resets. In point-process formulations, the dynamics may instead be specified through stochastic intensities or hazard functions driven by $\mu_{t}^{N}$.

In the mean-field regime of a large, weakly coupled network, one expects a law-of-large-numbers behavior as *N* → ∞: the empirical measure $\mu_{t}^{N}$ converges to a deterministic probability measure μ_*t*_ on *E*, and the dynamics of a typical neuron converges to a nonlinear (McKean-Vlasov) process *Z*(*t*) with law μ_*t*_,


$$\begin{array}{l} dZ\left(t\right)=b(Z\left(t\right),\mu_{t}(dt+\sigma )Z\left(t\right),\mu_{t}(dW\left(t\right)+dJ\left(t\right),\\ \;\mu_{t}=L\left(Z\left(t\right)\right). \end{array}$$

when *Z*(*t*) has continuous paths and a density *p*(*t, z*), its law solves a nonlinear Fokker-Planck or transport equation of McKean-Vlasov type. If some coordinates of *Z* are ages (time since last spike), or if jumps and reset mechanisms are present, transport terms and renewal-type boundary conditions appear, leading to age-structured or kinetic PDEs. Rigorous derivations rely on the theory of interacting particle systems and the propagation of chaos (Carrillo and Roux, 2025; Fasoli, 2013).

### 4.2 Fokker-Planck models

Fokker-Planck equations arise as forward Kolmogorov equations for stochastic neuron models and noisy neural-mass systems. They describe the time evolution of a density *p*(*t, x*) for a process


$$dX_{t}=b\left(X_{t},t\right)dt+\sigma \left(X_{t},t\right)dW_{t},$$

with $X_{t}\in {\mathbb{R}}^{d}$ encoding, for example, a membrane-potential or a low-dimensional rate/decision variable. If *X*_*t*_ admits a density, then *p* satisfies


$$\begin{array}{l} \partial_{t}p\left(t,x\right)=-\nabla_{x}\cdot (b\left(x,t\right)p\left(t,x\right))+\frac{1}{2}\nabla_{x}\cdot (a\left(x,t\right)\nabla_{x}p\left(t,x\right)),\;a=\sigma \sigma^{⊤}. \end{array}$$ (6)

when *b* or *a* depend on *p* through a self-consistent firing rate or mean-field, Equation 6 becomes a nonlinear Fokker-Planck equation of McKean-Vlasov type.

An important example outside the classical spiking setting is provided by Kuramoto-type oscillator networks, where population-level nonlinear Fokker-Planck equations have been used to describe synchronization phenomena in cortical oscillations (Breakspear et al., 2010).

#### 4.2.1 Network noisy leaky integrate-and-fire (NNLIF) models

In a homogeneous population of NNLIF neurons, let *I*_syn, *i*_(*t*) denote incoming synaptic current for neuron *i* at time *t*. Then, the subthreshold voltage dynamics of neuron *i* is governed by an SDE of the form


$$\begin{array}{l} dV_{i}\left(t\right)=\left(-\frac{1}{\tau_{m}}\left(V_{i}\left(t\right)-V_{\text{rest}}\right)+I_{\text{syn},i}\left(t\right)\right)dt+\sigma dW_{i}\left(t\right), \end{array}$$

with threshold and reset at *V*_th_ and *V*_reset_, respectively. In a mean-field approximation, *I*_syn, *i*_(*t*) is replaced by an effective input depending on the population firing rate *S*(*t*) and a connectivity parameter *J*, possibly with synaptic delay. In the limit, as *N* → ∞, the membrane-potential density *p*(*t, v*) satisfies a nonlinear Fokker-Planck equation in (*V*_min_, *V*_th_) of the form


$$\begin{array}{l} \partial_{t}p\left(t,v\right)+\partial_{v}\left(\left[-\frac{1}{\tau_{m}}\left(v-V_{\text{rest}}\right)+\mu \left(t\right)\right]p\left(t,v\right)\right)-\frac{\sigma^{2}}{2}\partial_{vv}^{2}p\left(t,v\right)=0, \end{array}$$

where drift μ(*t*) is determined by network activity *S*(*t*), and boundary conditions encode threshold crossing, a flux-based definition of the firing rate *S*(*t*), and reset at *V*_reset_.

An early analytical study of sparse inhibitory integrate-and-fire networks identified a sharp transition from stationary to oscillatory global activity in the low-firing-rate regime, providing a classical reference point for later population-level analyses of oscillations (Brunel and Hakim, 1999). Subsequent analytical studies of NNLIF equations have clarified several additional key regimes. The basic model admits zero, one, or two stationary states depending on connectivity, and in fully excitatory networks, one can prove finite-time blow-up of the firing rate under suitable conditions on the initial data, highlighting the delicate balance between excitation and noise (Cáceres et al., 2011). Extensions, including a refractory state and randomness in the firing potential, show that the blow-up scenario persists and that spontaneous activity can arise in parameter regimes where the deterministic-threshold model would blow up (Cáceres and Perthame, 2014). For inhibitory or weakly connected networks, and in particular for delayed NNLIF systems, one can construct global-in-time solutions and prove convergence toward the stationary distributions by exploiting a discrete sequence of pseudo-equilibria that captures the long-time behavior for large transmission delays (Cáceres et al., 2024). These results link the nonlinear Fokker-Planck structure of NNLIF models to phenomena such as plateau states and delay-induced oscillations in population activity.

#### 4.2.2 Fokker-Planck models for decision, rate, and evoked population dynamics

Fokker-Planck equations also arise for low-dimensional decision and rate models derived from stochastic neural-mass systems. In two-choice decision models, a stochastic Wilson-Cowan-type system with a drift field *F* and noise amplitude ε leads, at the level of probability density *p*(*t, x*) of the two population activities *x* ∈ Ω⊂ℝ^2^, to a Fokker-Planck equation of the form


$$\begin{array}{l} \partial_{t}p\left(t,x\right)=-\nabla_{x}\cdot \left(F\left(x\right)p\left(t,x\right)\right)+\frac{\varepsilon }{2}{\text{Δ}}_{x}p\left(t,x\right),\;x\in \text{Ω}\subset {\mathbb{R}}^{2}, \end{array}$$

supplemented with no-flux boundary conditions on ∂Ω that enforce bounded firing rates and conservation of total probability (Carrillo et al., 2011). For small noise, the dynamics exhibit metastability: solutions rapidly concentrate along a slow manifold connecting an unstable “spontaneous” state to two stable “decision” states, and only on much longer time scales relax toward equilibrium. Under suitable inward-pointing assumptions on the drift field *F*, one can prove the existence, uniqueness, and positivity of a stationary density and convergence of solutions toward this equilibrium by combining Krein-Rutman theory with generalized relative entropy methods (Carrillo et al., 2011). The stationary distribution encodes decision probabilities through the mass near the different attractors, and the observed concentration along a slow manifold suggests reduced one-dimensional drift-diffusion approximations for the decision variable (Carrillo et al., 2011).

Furthermore, in (Harrison et al. 2005), a population density approach is proposed to model the evoked response potential with random arrivals at peak times, and the dispersive effects of stochastic forces are analyzed to compare equilibrium response rates of stochastic versus deterministic models. Taking expectations over the probability density that represents an ensemble of trajectories, the mean membrane potential and firing rates are studied. Model parameters are estimated via a Bayesian approach, and the corresponding Fokker-Planck equations are solved via discretizations.

### 4.3 Age- and time-elapsed structured PDEs

In computational neuroscience, age-structured and time-elapsed formulations, often referred to as refractory-density equations, provide an alternative mean-field description in which the state variable is the time-elapsed since the last spike rather than the membrane-potential (Chizhov and Graham, 2007, 2008; Gerstner, 2000, 2001; Schwalger and Chizhov, 2019). At the microscopic level, each neuron carries an age variable *A*_*i*_(*t*) ≥ 0 that increases at unit speed between spikes and is reset to zero at spike-times,


$$\begin{array}{l} {Ȧ}_{i}\left(t\right)=1, \end{array}$$

with spike intensity (hazard rate) $\lambda \left(A_{i}\left(t\right),\mu_{t}^{N}\right)$ depending on age and population activity. In the mean-field limit, the age distribution *n*(*t, a*) satisfies


$$\begin{array}{l} \partial_{t}n\left(t,a\right)+\partial_{a}n\left(t,a\right)+\text{λ}\left(a,F\left[n\left(t,\cdot \right)\right]\right)n\left(t,a\right)=0,\;a>0, \end{array}$$

together with the renewal boundary condition


$$\begin{array}{l} n\left(t,0\right)=\int_{0}^{\infty }\text{λ}\left(a,F\left[n\left(t,\cdot \right)\right]\right)n\left(t,a\right)da. \end{array}$$

These equations naturally encode refractoriness through the age variable and can be extended to incorporate adaptation and fatigue through fragmentation-type terms, as well as transmission delays through suitable coupling kernels (Pakdaman et al., 2009, 2014; Perthame et al., 2025). Analytical studies of age- and time-elapsed structured models have shown that, depending on the connectivity regime and on heterogeneity or adaptation mechanisms, one can observe rigorous desynchronisation and exponential relaxation to steady states in weakly coupled or heterogeneous networks (Kang et al., 2015; Pakdaman et al., 2009, 2014), as well as self-sustained or delay-induced periodic solutions in strongly nonlinear and delayed regimes (Pakdaman et al., 2009; Perthame et al., 2025).

### 4.4 Kinetic PDEs for voltage-conductance and FitzHugh-Nagumo dynamics

Kinetic mean-field models retain several continuous state variables per neuron, such as a membrane-potential and one or more conductance or recovery variables, together with noise and mean-field coupling. They aim to bridge biophysically detailed single-neuron dynamics with more aggregated population models.

A paradigmatic example is the voltage-conductance kinetic equation introduced by Perthame and Salort for integrate-and-fire networks with slow synaptic conductances (Perthame and Salort, 2013). In the mean-field limit the joint density *p*(*t, v, g*) of voltage *v* and conductance *g* satisfies a kinetic Fokker-Planck equation of the form


$$\begin{array}{l} \partial_{t}p\left(t,v,g\right)+\partial_{v}(f\left(v,g\right)p(+\partial_{g}(h\left(v,g,\text{Φ}\left[p\right]\right)p)-\frac{\sigma_{V}^{2}}{2}\partial_{vv}^{2}p\\ \;-\frac{\sigma_{G}^{2}}{2}\partial_{gg}^{2}p=0, \end{array}$$

where *f* and *h* encode the passive and synaptic currents, Φ[*p*] is a nonlocal functional of *p* linked to the population firing rate, and the diffusion term acts only on the conductance variable *g*, yielding a hypoelliptic structure. The equation is complemented by threshold-reset conditions in *v* and suitable boundary behavior as *g* → 0 and *g* → ∞. Perthame and Salort established global a priori bounds on the density and the firing rate, analyzed the existence of stationary solutions in weakly connected regimes, and highlighted a “paradox” with respect to parabolic NNLIF equations, which can exhibit finite-time blow-up whereas the kinetic model remains globally bounded (Perthame and Salort, 2013).

Another prominent example is the kinetic FitzHugh-Nagumo model, in which the probability density of the state (*V, W*) satisfies a nonlinear Fokker-Planck equation associated with mean-field-coupled FitzHugh-Nagumo dynamics with noise (Mischler et al., 2016). The associated density *p*(*t, v, w*) satisfies a hypoelliptic nonlocal kinetic Fokker-Planck equation on ℝ^2^, where the drift combines the cubic FitzHugh-Nagumo vector field with a McKean-Vlasov term depending on the average voltage. Mischler et al. proved the global well-posedness of this equation, the existence of non-trivial stationary probability densities, and uniqueness together with exponential stability of the stationary state in a weakly nonlinear (small connectivity) regime (Mischler et al., 2016). Moreover, their spectral analysis and numerical simulations show that increasing connectivity can induce symmetry breaking and give rise to time-periodic collective oscillations, illustrating how kinetic PDEs capture transitions between asynchronous and oscillatory network activity (Mischler et al., 2016).

### 4.5 Stochastic neural field PDEs

Neural field models describe coarse-grained activity in spatially distributed neuronal populations, typically cortical sheets or grid-cell networks. Classical formulations are integro-differential equations for a field *u*(*t, x*) defined on a spatial domain, with nonlocal coupling via a synaptic kernel. A classic review of deterministic Wilson-Cowan/Amari-type neural field theories, including their mathematical analysis and pattern-forming mechanisms, is given by Coombes (Coombes, 2005).

Building on these classical formulations, stochastic neural field models incorporate noise either directly at the field level or via fluctuations in local populations and exhibit spatial patterns such as traveling waves, bumps, and grid-like tessellations. From a mean-field PDE perspective, stochastic neural fields can often be recast as nonlinear Fokker-Planck systems for the local probability density of activity variables. Recent work in (Carrillo et al. 2023, 2024) develops this framework for spatially coupled neural populations with stochastic dynamics.

In (Carrillo et al. 2023), they analyze a four-population model for grid cells in the medial entorhinal cortex on a toroidal domain, in which spatially periodic patterns emerge from a homogeneous state through a noise-driven bifurcation: small but finite noise destabilizes the spatially homogeneous equilibrium and selects hexagonal grid-like patterns of activity, in line with experimental observations. The analysis combines neural-field pattern-formation mechanisms with a nonlinear, nonlocal Fokker-Planck structure and uses equivariant bifurcation theory on the torus to classify branches of patterned solutions and their linear stability (Carrillo et al., 2023).

Building on this model, (Carrillo et al. 2024) develops a rigorous PDE theory for the associated nonlinear Fokker-Planck system. By reformulating the equation as a Stefan-type free boundary problem, the authors prove the local and global existence of classical solutions under suitable decay and Lipschitz assumptions, derive representation formulas, and establish nonlinear asymptotic stability of stationary states via a generalized relative entropy functional. These results complement the bifurcation analysis of (Carrillo et al. 2023) by showing that the spatially structured grid-cell patterns that emerge at the PDE level are dynamically stable over long times.

Beyond neural field Fokker-Planck systems, SPDEs have also been used to model cortical activity more directly. For example, Steyn-Ross- type SPDE models are used in (Kramer et al. 2005) to predict oscillations and traveling waves in response to the increased subcortical excitation, with a simplified ODE version showing bifurcations and oscillatory behavior. Comparison with human seizure data reveals agreement in peak frequency and propagation speed, suggesting that seizures may represent the formation of a pathological pattern in the brain (Kramer et al., 2005). Another example is a feedback control model of (Lopour and Szeri 2010) via an SPDE model of the human cortex for stopping seizures. It ensures accurate simulations by handling sharp changes in the brain's activity near electrodes. It is shown that a new electrode measurement model and control algorithm effectively suppress seizure-like activity while minimizing the risk of cortical damage (Lopour and Szeri, 2010).

A numerical solution method for stochastic Fitzhugh-Nagumo PDEs is developed in (Uma et al. 2025) for modeling neuro-biological dynamics as


$$\begin{array}{l} u_{t}=u_{xx}+u\left(u-a\right)\left(1-u\right)+\sigma u\dot{W_{t}} \end{array}$$

with initial and boundary conditions


$$\begin{array}{l} u\left(0,x\right)=u_{0}\left(x\right),0\leq x\leq 1\\ u\left(t,0\right)=g_{0}\left(t\right),u\left(t,1\right)=g_{1}\left(t\right),0\leq t\leq T \end{array}$$

where *W*_*t*_ is a Wiener process and Ẇ is the corresponding white noise process.

To summarize, mean-field PDE models in neuroscience encompass parabolic, hyperbolic, and kinetic equations that systematically connect microscopic stochastic descriptions of neural networks to mesoscopic and macroscopic dynamics and provide a flexible framework for analyzing population-level neural phenomena.

### 4.6 Numerical methods for mean-field PDE models

The mean-field PDE models described in this section are rarely solvable in closed form and are typically investigated using numerical schemes tailored to their specific structure. Two broad approaches can be distinguished: particle-based methods, which approximate the underlying stochastic processes, and deterministic solvers for the PDEs themselves.

A first class of methods relies on Monte Carlo simulation of the microscopic SDE or point-process description and empirical estimation of the associated density (René et al., 2020; Schwalger et al., 2017). This is natural for NNLIF and kinetic models, where the law of a McKean-Vlasov process is approximated by a large ensemble of interacting particles. Such schemes are straightforward to implement but can be computationally expensive when rare events or fine spatial resolution are required, and are less convenient for systematic bifurcation analysis.

Deterministic schemes approximate the PDEs directly. For the NNLIF equation and its variants, finite-difference and finite-volume methods based on high-order upwind or WENO discretisations have been used to resolve sharp gradients and blow-up-like phenomena in voltage or conductance distributions, often combined with strong-stability-preserving Runge-Kutta time integrators. In addition, structure-preserving discretisations of Fokker-Planck type, such as the Chang-Cooper and Scharfetter-Gummel schemes, have been adapted to these models in order to guarantee discrete conservation of mass and non-negativity of the solution and, in the linear case, to reproduce the correct equilibrium state and dissipation of relative entropy (Carrillo and Roux, 2025; Chang and Cooper, 1970).

For age-structured and time-elapsed equations, upwind finite-volume methods on the age variable, sometimes coupled with splitting strategies in time, provide efficient approximations of transport and renewal terms. In kinetic voltage-conductance and FitzHugh-Nagumo systems, semi-implicit and operator-splitting schemes are often employed to handle stiffness and degenerate diffusion, while maintaining stability in regimes with strong coupling or large delays.

More recently, spectral and discontinuous Galerkin methods, as well as multiscale hybrid schemes combining particle and PDE solvers, have been proposed for stochastic neural field PDEs and related Fokker-Planck systems. These approaches aim to accurately capture both the spatial pattern formation and the long-time dynamics (including noise-driven bifurcations) in high-dimensional settings. Developing robust, structure-preserving, and computationally efficient numerical methods for these PDE models remains an active area of research and are crucial to connecting the mathematical theory to realistic network sizes and experimental data.

## 5 Discussion and open problems

In this review, we have organized differential-equation-based models in neuroscience into three interconnected layers: deterministic ODE models at the single-neuron, population and whole-brain scales; stochastic models that extend these ODE frameworks to incorporate intrinsic and synaptic variability; and mean-field PDE models that describe the evolution of probability or population densities in large networks (Carrillo and Roux, 2025; Dayan and Abbott, 2001; Ermentrout and Terman, 2010; Gerstner et al., 2014). This hierarchy links microscopic spiking to mesoscopic and macroscopic behavior and reveals common mathematical structures across modeling approaches.

At the deterministic level, conductance-based and reduced single-neuron models, neural-mass systems and whole-brain ODE networks provide mechanistic templates from which both stochastic and mean-field descriptions can be derived (Breakspear, 2017; Deco et al., 2008; FitzHugh, 1961; Hodgkin and Huxley, 1990; Ritter et al., 2013; Wilson and Cowan, 1972). They allow one to analyze excitability types, multistability and oscillations in relatively low-dimensional state spaces, to connect biophysical parameters to qualitative changes in dynamics, and to interpret mesoscopic and macroscopic recordings via fitted population models. Stochastic extensions (noisy ODEs, SDEs, point-process and renewal models) enrich these templates by capturing randomness in spike-timing, synaptic input and network connectivity, and they serve as microscopic starting points for mean-field limits. Because variability and noise are central to neural function and cognition, the stochastic layer receives particular attention in this review. SDE-based models have been successfully used to reproduce neural firing patterns, to study the impact of fluctuating inputs and synaptic plasticity, and to capture large-scale neural variability in electro-physiological (Ditlevsen and Samson, 2016; Jansen and Rit, 1995; René et al., 2020; Schwalger et al., 2017) and imaging data (Jirsa et al., 2017; Ritter et al., 2013).

On the other hand, mean-field PDE models-Fokker-Planck, age-structured, kinetic and stochastic neural field equations-encode the collective dynamics of large populations in terms of densities over membrane-potentials, elapsed times or conductance and recovery variables (Cáceres et al., 2011; Carrillo and Roux, 2025; Chizhov and Graham, 2007; Gerstner, 2000; Mischler et al., 2016; Pakdaman et al., 2013; Schwalger et al., 2017). Across these levels, similar dynamical phenomena emerge: multistability, oscillations, synchronization and pattern formation, as well as regimes of pathological activity such as seizure-like events, blow-up and delay-induced instabilities. Taken together, ODE, SDE and PDE models have made significant contributions to our understanding of neural dynamics and remain promising tools for advancing our understanding of brain function, neural disorders and the development of novel computational methods in neuroscience.

Although the basic mathematical picture is well established for several canonical models, many questions remain open. At the stochastic level, network-scale SDE models are often high-dimensional and driven by complex, state-dependent and non-Gaussian noise sources, yet most existing work still relies on relatively simple white-noise approximations. Developing statistically tractable and computationally efficient methods to infer realistic noise structures and parameters from limited, noisy data (e.g. subsampled spikes or imaging signals) remains a major challenge. Recent work on neural ODE and SDE architectures has also emphasized open problems concerning their expressive power, identifiability and numerical robustness: it is not yet fully understood which classes of neural dynamics these models can represent, how to ensure stable training in stiff and multiscale regimes, or how to interpret the learned continuous-time vector fields in biological terms (Worsham and Kalita, 2025). From a methodological perspective, it would be valuable to design parameter-estimation and model-selection frameworks that combine mechanistic differential equation models with modern machine-learning tools (e.g. amortized inference, variational autoencoders, or neural surrogate models), in a way that is robust to measurement noise, yields uncertainty quantification, and respects biophysical constraints.

Similar challenges arise when combining differential equation models with machine-learning tools such as physics-informed neural networks (PINNs). PINN-based methods have been proposed for forward and inverse problems in Fokker-Planck and mean-field PDEs, but rigorous error estimates, convergence guarantees and identifiability results are still largely restricted to low-dimensional or linear settings. Extending these theoretical foundations to nonlinear, high-dimensional neural PDEs, and understanding how much data are needed to learn reliable models from particle or spike-train observations, are important open questions. More broadly, there is considerable scope for work on multiscale integration-connecting ODE, SDE and PDE descriptions within unified inference pipelines-on control and optimization of high-dimensional stochastic and mean-field models, and on incorporating more realistic biophysical constraints and noise statistics into differential-equation-based models of neural systems.

At this point, we believe that artificial intelligence and machine-learning techniques may enable more precise and efficient parameter estimation for stochastic neural models. Since these approaches can learn complex probabilistic structure directly from data, they have the potential to substantially accelerate parameter estimation, especially when combined with adaptive optimization and uncertainty quantification. However, a key challenge is to ensure that the inferred parameters remain biologically interpretable and robust in real-data settings.

### 5.1 Multiscale integration and model comparison

Although ODE-/SDE-/PDE-based models can often be derived from one another in suitable limiting regimes, systematic cross-scale comparisons within the same study are not yet widespread, despite influential examples that provide extensive benchmarks between derived mesoscopic/macroscopic dynamics and simulations of the underlying microscopic models (Greven et al., 2026; René et al., 2020; Schwalger et al., 2017). Beyond these benchmark studies, many correspondences are known at the level of formal derivations: for instance, NNLIF Fokker-Planck equations and time-elapsed age-structured models can both be obtained from networks of integrate-and-fire neurons under different scalings or choices of state variables (Cáceres et al., 2024, 2011; Carrillo et al., 2011); kinetic FitzHugh-Nagumo equations can be linked to both microscopic excitable neurons and low-dimensional rate models (Mischler et al., 2016; Montbrió et al., 2015). A more systematic analysis of these correspondences could clarify when different reduced descriptions are genuinely equivalent, when they capture complementary aspects of the same dynamics, and when they diverge.

From a practical point of view, such comparisons are also important for model selection: given a particular dataset (e.g., spike trains, local field potentials, EEG/MEG or fMRI time series), under what conditions should one prefer a neural-mass ODE, a Fokker-Planck equation, an age-structured model or a kinetic PDE? Developing quantitative criteria and robust procedures for comparing these models, either through likelihood-based approaches or via carefully designed summary statistics, remains an open challenge.

### 5.2 Data assimilation and parameter inference in PDE-based models

While there is a wide variety of well-established methods for parameter inference in ODE and SDE models, robust and identifiable inference is still nontrivial in multiscale, partially observed, and strongly nonlinear neural systems (Ditlevsen and Samson, 2016; Moye and Diekman, 2018; René et al., 2020). For PDE-based neural models, corresponding inference and data-assimilation frameworks are comparatively less standardized and less routinely applied in neuroscience (Harrison et al., 2005). In principle, population densities in Fokker-Planck, age-structured or kinetic equations can be linked to observable quantities such as firing rates, power spectra or spatial patterns; in practice, the inverse problem of estimating parameters (connectivity strengths, delays, noise levels, adaptation time scales) from such observables remains highly challenging (Ditlevsen and Samson, 2016; Harrison et al., 2005; René et al., 2020).

Several methodological avenues are available, such as variational data assimilation, adjoint-based gradient methods, Bayesian inference, and particle-based approximations of PDE dynamics, but they have not yet been systematically adapted to neural PDE models. Developing scalable, well-posed parameter-estimation frameworks for these equations and quantifying the identifiability of key parameters is an important direction for making mean-field PDEs more directly usable in data-driven neuroscience.

### 5.3 Control, modulation and optimal interventions

Most of the work reviewed here focuses on the autonomous dynamics of neural systems. However, from both theoretical and applied perspectives, questions of control and modulation are increasingly relevant, for example, in the context of neuromodulation therapies, seizure suppression, or targeted stimulation (Acharya et al., 2022; Chouzouris et al., 2021; Schiff, 2011).

At the ODE level, there is a rich literature on control of neural-mass and whole-brain models (Basu et al., 2018; Chouzouris et al., 2021; Wang et al., 2016). Moreover, stochastic optimal control techniques (based on dynamic programming principle or martingale duality techniques) are well established (Chen et al., 2017). Even though there may still be some room to extend currently known methods to interventions in neurodynamics, there is an increasing trend to integrate theoretical results with both machine-learning and Monte Carlo methods. However, control questions for PDE-based mean-field models are only beginning to be explored. Natural problems include: suppressing blow-up in NLIF equations by appropriately shaping external input or connectivity; steering age-structured networks between asynchronous and oscillatory regimes; and controlling kinetic equations to stabilize or destabilize particular patterns. These tasks naturally lead to optimal control or feedback control problems governed by nonlinear Fokker-Planck, transport, or kinetic equations, where control theory and numerical analysis still have much to contribute.

### 5.4 Analytical challenges and model generalizations

On the analytical side, several structural questions remain open even for relatively classical models (Carrillo and Roux, 2025). For NNLIF equations, a complete characterization of all possible long-time behaviors (stationary states, periodic or more complex attractors) as connectivity, delay, and noise vary is still incomplete, especially in multi-population or spatially extended settings (Cáceres et al., 2011; Cáceres and Perthame, 2014; Cáceres et al., 2024). For age-structured models, strongly nonlinear firing-rate functions and distributed delay kernels can generate intricate dynamics whose rigorous understanding is only partial (Pakdaman et al., 2009, 2013, 2014; Perthame et al., 2025; Schwalger and Chizhov, 2019). For kinetic equations, hypoellipticity, degenerate diffusion, and unbounded coefficients pose technical difficulties, and the study of bifurcations and pattern formation is still in an early stage (Mischler et al., 2016; Perthame and Salort, 2013).

At the same time, several natural model extensions have not yet been fully incorporated into the mean-field PDE framework. These include synaptic plasticity (both short- and long-term), structural connectivity changes, heterogeneous populations with distributed parameter sets, and more realistic dendritic and network geometries (Carrillo et al., 2023; Ritter et al., 2013; Robert and Vignoud, 2021; Schmutz et al., 2023; Schwalger et al., 2017). Extending existing analytical results to such generalizations or identifying reduced PDE descriptions that remain tractable in their presence, represents a substantial but important challenge.

### 5.5 Links to machine-learning and neural differential equations

Finally, there is growing interest in combining mechanistic differential equation models with machine-learning techniques. At the ODE and SDE levels, neural ODEs, neural SDEs and universal differential equations offer flexible frameworks in which parts of the dynamics are specified mechanistically while others are learned from data. However, combining these models with Bayesian and statistical methods, such as (conditional) maximum-likelihood estimation and EM-type approaches within hidden Markov frameworks, becomes challenging, especially for mesoscopic inference when some variables are latent and only partially observed (Ditlevsen and Samson, 2016; Laing and Lord, 2009; René et al., 2020). Extending these ideas to PDE-based neural models could enable hybrid approaches where, for example, the structure of a Fokker-Planck or age-structured equation is retained but certain closure terms, effective drift components, or kernel functions are learned from data.

Physics-informed neural networks and related mesh-free methods provide another route to approximate solutions of neural PDEs and to solve inverse problems, potentially reducing the cost of classical discretisations in high-dimensional or parameter-rich settings. However, questions of expressivity, training stability, and error control for such methods in the context of nonlinear Fokker-Planck, transport, and kinetic equations are far from settled, and deserve careful investigation.

### 5.6 Outlook

Differential equation models have played a central role in theoretical neuroscience for decades, and the landscape is now broad enough to cover deterministic dynamics, stochastic processes, computational methods and mean-field PDEs in a unified way. The next phase is likely to be driven by tighter integration of these modeling layers with experimental data and by closer interaction between theoretical analysis, numerics/simulations, optimization/control, and machine-learning components. Progress along these lines could yield not only a deeper mathematical understanding of neural dynamics across scales, but also more effective tools for interpreting data, designing interventions, and linking microscopic mechanisms to mesoscopic and macroscopic brain activities.
